# The Updated Assessment of the Liverwort Flora of Laos, the Least-Studied Higher Plants Group in Indochina

**DOI:** 10.3390/plants14243832

**Published:** 2025-12-16

**Authors:** Vadim A. Bakalin, Seung Se Choi, In Chun Hwang, Myung-Ok Moon, Ksenia G. Klimova

**Affiliations:** 1 Laboratory of Cryptogamic Biota, Botanical Garden-Institute FEB RAS, Makovskogo Street 142, Vladivostok 690024, Russia; ksenia.g.klimova@mail.ru; 2 Team of National Ecosystem Survey, National Institute of Ecology, Seocheon 33657, Republic of Korea; 3 Department of Life Science, Jeonbuk National University, Jeonju 54896, Republic of Korea; plant836@nate.com; 4 Institute of Forestree, Jeju 63133, Republic of Korea; egosari@naver.com

**Keywords:** Hepaticae, Southeast Asia, diversity, distribution patterns, ecology, altitudinal patterns

## Abstract

The previously published liverwort checklist of Laos, one of the least-studied countries in Asia, was titled “Listing the Unknown”, based on the fact that only 66 species are known for such a landscape-diverse country. Our collection revealed 39 genera and 76 species, 62 of which are newly recorded species to the country, bringing the total number of known species to 128. Among the reported genera, there are 22 liverwort genera new to Laos, all of which could have been expected in this area. Although new data expands the species list, the total number of species recorded remains inadequately small. The presented studies are based primarily on collections at lower elevations (below 500 m above sea level), in strongly modified secondary forest conditions, and are of interest specifically as an example of the liverwort flora of heavily modified, anthropogenically disturbed habitats of rather dry tropical forest communities. The provided checklist includes data on the ecological conditions of the collected species and their altitudinal range. Further research on the liverwort flora of Laos should be conducted in the upper altitudinal zones of the north and the east of the country.

## 1. Introduction

The Southeast Asian mainland, roughly equivalent to the Indochina Peninsula in a geographical sense and the Indochina Floristic Region [1], has long attracted the attention of botanists. If we were to exclude the Malay Peninsula, which belongs to the Malesian Floristic Region [1] and is characterized by different flora and a more humid climate, the Indochina Region encompasses five countries: part of Myanmar, all of Thailand, Vietnam, Cambodia, and landlocked Laos. Thailand has long been the most studied country in liverwort research, in large part owing to the efforts of Dr. Naofumi Kitagawa [2,3,4,5,6,7,8,9], who devoted several papers to the liverwort flora of the country. These papers provided us with data on what can be expected in other areas of the Indochina Peninsula. Data on its liverwort and hornwort diversity were summarized by Lai et al. [10]. Vietnam, for which the first list of liverworts was completed in 1965 [11], began to compete with Thailand in liverwort knowledge in the mid-20th century. The cited checklist, while a milestone in the study of its flora, also served as a stimulus for new research, as the number of species listed (165) was clearly inconsistent with the presumed reality. Dr. Pócs, who has published (and continues to publish) numerous studies devoted to both taxonomic revisions of some groups and additions to the total list of the Vietnamese liverwort flora [12,13,14,15,16,17,18,19,20], is primarily the key figure in advancing knowledge of the Hepaticae of Vietnam. In 2016 and 2017, the new checklists of Vietnamese liverworts were published [21,22]. Taking into account later additions [23,24,25,26,27,28,29,30,31,32,33,34,35,36,37,38], over 600 species are now known in Vietnam liverwort flora.

Far less attention has been paid to the liverwort flora of the Indochina ‘middle axis’ countries—Cambodia and Laos. For both, information is scattered across publications devoted to new reports and taxonomic revisions for larger territories. However, Cambodia is more fortunate in this regard, as it has a number of targeted studies conducted by Tixier [39,40,41,42,43,44,45], including a description of the unique South Indochina tropical liverwort flora of Mount Bokor [43]. A list of Cambodian liverworts was first published by a team led by Ingerpuu et al. [46], providing data on the distribution of 138 species. A summary of the data collected by our team (far from complete) enabled us to document an additional 66 species, including representatives of 20 genera new to the country [47]. One species was added later [28]. Both studies demonstrated how little we still know about the liverwort inhabitants of this country. However, the situation with Laos is even more problematic. Only 66 liverwort species and not a single hornwort are known there [48].

In the first Laos liverwort checklist, Söderström et al. [48] provided a map of Laos biomes and ecoregions and data on the state of knowledge regarding liverwort diversity. The attempts to make some progress in understanding the species diversity of Laos, given that almost nothing is known about the country’s liverwort diversity, have so far been rather limited. However, we believe it is justified to present new information on the liverworts of Laos, which is the aim of this account.

## 2. Results

### 2.1. General Statistics

A total of 189 specimens were studied in the course of the present work. Within the specimens, 224 identifications were made, as some specimens were represented by a mixture of species. The identification process revealed 22 genera new to the Laos liverwort flora: *Asperifolia* A.V. Troitsky, Bakalin & Maltseva, *Calycularia* Mitt., *Calypogeia* Raddi, *Ceratolejeunea* (Spruce) J.B. Jack & Steph., *Cyathodium* Kunze ex Lehm., *Dumortiera* Nees, *Herbertus* Gray, *Kurzia* G. Martens, *Lepidolejeunea* R.M. Schust, *Lopholejeunea* (Spruce) Steph., *Marchantia* L., *Mastigolejeunea* (Spruce) Steph., *Microlejeunea* (Spruce) Steph., *Notoscyphus* Mitt., *Pallavicinia* Gray, *Plagiochila* (Dumort.) Dumort., *Pleurozia* Dumort., *Plicanthus* R.M. Schust., *Riccardia* Gray, *Scapania* (Dumort.) Dumort., *Schiffneriolejeunea* Verd. and *Schistochila* Dumort.

A total of 76 species were identified in the collection. Of these, 62 (thus ca. 80%) are new to the flora of Laos, bringing the total number of species known to the country to 122. The newly recorded taxa are marked with an asterisk in the following list.

The number of species identified by province is as follows: Phongsaly Province—2 species; Oudomxay Province—1 species; Vientiane Province—4 species; Xaisomboun Province (as a part of Xiangkhouang Province)—20 species; Bolikhamsai Province—41 species; Khammouane Province—7 species; Champasak Province—9 species.

### 2.2. List of Species

*Acrolejeunea recurvata* Gradst.—**15**, **17**—317, 354 m—Evergreen tropical forest in a broad stream valley: open dry bark of living standing tree trunk. Evergreen secondary tropical forest along a sluggishly flowing stream in abroad valley: partly shaded dry boulder near the stream—Associates: *Frullania motoyana*—L-1-6-24, L-6-38-24.

*Asperifolia indosinica Bakalin et A.V. Troitsky—**9**—280 m—Evergreen secondary tropical forest with tall bamboo understory along a sluggishly flowing stream: partly shaded moist clayish soil on steep slope to stream—Associates: *Dumortiera hirsuta*, *Heteroscyphus coalitus*, *Pallavicinia levieri*—L-5-10a-24.

*Bazzania callida (Sande Lac. ex Stephani) Abeyw.—**8**, **9**, **10**, **17**, **18**—280–381 m—Evergreen secondary tropical forests along sluggishly flowing streams: open dry cliff near the stream, partly shaded mesic clayish soil on the trail, partly shaded mesic soil on steep slopes—Associates: *Dumortiera hirsuta*, *Pallavicinia levieri*—L-5-4-24, L-5-10-24, L-5-12-24, L-5-21-24, L-5-22-24, L-6-11-24, Lao24055a, Lao24055c, Lao24057a, Lao24102—Comment: the morphological deviations are described in a separate paper devoted to Lepidoziaceae updates in East Indochina (Bakalin et al., 2026, in preparation).

*Bazzania oshimensis (Stephani) Horik.—**17**—354 m—Evergreen secondary tropical forest along a sluggishly flowing stream in wide valley: partly shaded mesic boulder—L-6-2-24—Comment: The status of the taxon is questionable as discussed in [47] and requires further verification.

*Bazzania siamensis (Steph.) Bakalin et Klimova—**4**—1805 m—No habitat data are provided in the labels; based on satellite imagery, this area represents a very scattered secondary forest on a gentle slope—Associates: *Plagiochila beddomei*—2023-24, 2023-25.

*Bazzania tridens* (Reinw., Blume et Nees) Trevis.—**16**, **17**, **18**—354–381 m—Evergreen secondary tropical forests along sluggishly flowing streams in wide valleys: partly shaded dry clayish soil on steep slope—L-6-1-24, Lao24091, Lao24105, Lao24124.

*Calycularia crispula Mitt.—**5**—2690 m—Evergreen montane rain forest on slope: partly shaded to open, moist to mesic cliff near the valley—Lao25195.

**Figure 1:**
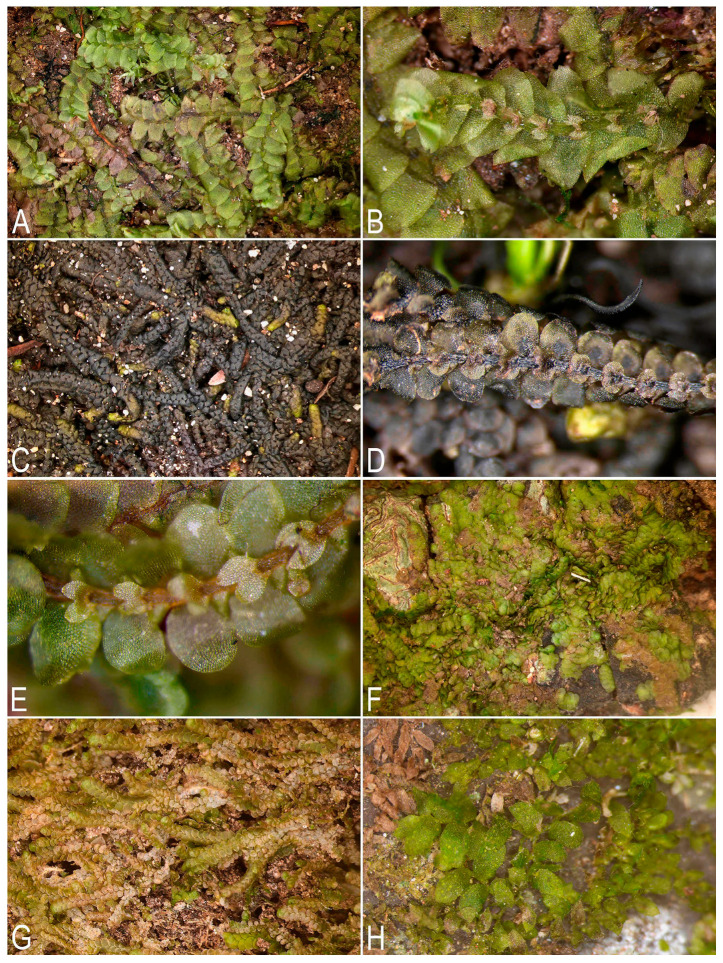
Some liverworts newly recorded in Laos (photos were made using a dissecting microscope in the laboratory): (**A**,**B**) *Calypogeia tosana* (Steph.) Steph.; (**C**,**D**) *Ceratolejeunea singapurensis* (Lindenb.) Schiffn.; (**E**) *Cheilolejeunea rigidula* (Nees ex Mont.) R.M. Schust; (**F**) *Cheilolejeunea ryukyuensis* Mizut.; (**G**) *Cheilolejeunea trifaria* (Reinw., Blume et Nees) Mizut.; (**H**) *Cololejeunea gottschei* (Stephani) Mizut.; (**A**,**B**) from L-6-15-24, (**C**,**D**) from L-6-32-24, (**E**) from L-6-19-24, (**F**) from L-5-14-24, (**G**) from L-1-3-24, (**H**) from L-6-9-24 (VBGI) (photos by V.A. Bakalin, 2024).

*Calypogeia tosana (Steph.) Steph.—**17**, **18**—354, 381 m—Evergreen secondary tropical forests along sluggishly flowing streams in wide valleys: partly shaded mesic clayish soil near the stream—L-6-15-24, Lao24125. Figure 1A,B.

*Caudalejeunea reniloba* (Gottsche) Stephani—**17**, **18**—354, 381 m—Evergreen secondary tropical forests along sluggishly flowing streams in wide valleys: partly shaded dry leaves of living shrubs—L-6-42-24, L-6-44-24, Lao24095.

*Ceratolejeunea belangeriana (Gottsche) Stephani—**26**—973 m—No habitat data are provided in the 2023-39 label; due to data from satellite photographs, there is a rather dense evergreen forest along a wide stream valley—Associates: *Lejeunea discreta*, *Spruceanthus semirepandus*—2023-39.

*Ceratolejeunea singapurensis (Lindenb.) Schiffn.—**14**, **16**, **17**—313–379 m—Evergreen tropical and evergreen secondary tropical forests in wide stream valleys: partly shaded dry boulders near the stream—L-6-32-24, L-6-34-24, Lao24001, Lao24003, Lao24080, Lao24081—Comment: Mizutani (p. 309) [49] wrote “this species is closely related to *Ceratolejeunea oceanica* [=*C. belangeriana*, V.A.B.], or may be an extreme form of *C. oceanica*”. The Catalogue of Life (https://www.catalogueoflife.org/, accessed on 10 December 2025) and World liverwort checklist [50] maintain both taxa at the species status that we are following for this concept, but we consider further studies as necessary to resolve this issue. Figure 1C,D.

*Cheilolejeunea rigidula (Nees ex Mont.) R.M. Schust. (=*Cheilolejeunea serpentina* (Mitt.) Mizut.)—**16**, **17**—354, 379 m—Evergreen secondary tropical forest along sluggishly flowing stream in wide valley: partly shaded dry bark of living tree branch, partly shaded dry boulder near the stream, partly shaded dry cliff—L-6-19-24, L-6-21-24, L-6-30-24, L-6-33-24, Lao24072—As it is accepted in Catalogue of Life (https://www.catalogueoflife.org/data/taxon/TS6K, accessed on 10 December 2025), *C. serpentina* is treated as a synonym of *C. rigidula*. Figure 1E.

*Cheilolejeunea ryukyuensis Mizut.—**8**, **9**, **10**—280–333 m—Evergreen secondary tropical forest with tall bamboo understory along sluggishly flowing stream: partly shaded dry bark of living tree branch—L-5-14-24, Lao24051, Lao24063. Figure 1F.

*Cheilolejeunea trapezia* (Nees) Kachroo et R.M. Schust.—**16**, **17**, **19**—184–379 m—Evergreen tropical and evergreen secondary tropical forests in broad stream valleys: partly shaded dry bark of living tree trunks—Associates: *Lepidolejeunea bidentula*—L-6-26-24, Lao24040, Lao24085.

*Cheilolejeunea trifaria (Reinw., Blume et Nees) Mizut.—**15**—317 m—Evergreen tropical forest in broad stream valley: partly shaded mesic tree trunk—Associates: *Lejeunea* sp., *Schiffneriolejeunea polycarpa*—L-1-3-24, L-1-4-24, L-1-4a-24. Figure 1G.

**Figure 2:**
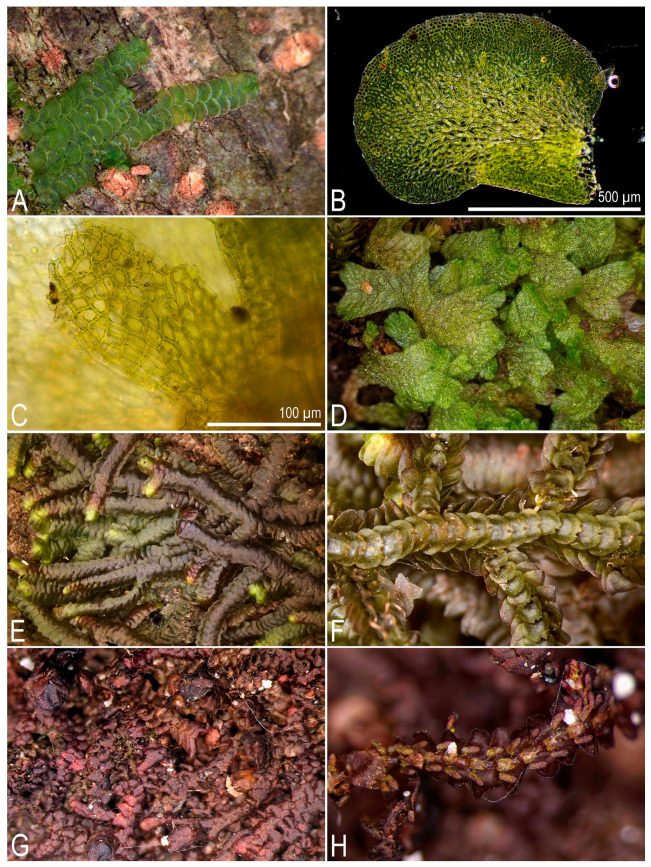
Some liverworts newly recorded in Laos (photos were made using upright compound and dissecting microscopes in the laboratory): (**A**,**B**) *Cheilolejeunea vittata* (Stephani ex G. Hoffm.) R.M. Schust. et Kachroo; (**C**) lobule of *Cololejeunea raduliloba* Stephani; (**D**) *Cyathodium* cf. *cavernarum* Kunze; (**E**,**F**) *Frullania gaudichaudii* (Nees et Mont.) Nees et Mont.; (**G**,**H**) *Frullania motoyana* Steph.; (**A**,**B**) from L-6-22-24, (**C**) from L-5-19-24, (**D**) from L-4-2-24, (**E**,**F**) from L-1-2-24, (**G**,**H**) from L-1-5-24 (VBGI) (photos by V.A. Bakalin, 2024).

*Cheilolejeunea vittata (Stephani ex G. Hoffm.) R.M. Schust. et Kachroo—**17**—354 m—Evergreen secondary tropical forest along a sluggishly flowing stream in broad valley: partly shaded dry bark of living tree trunk—L-6-22-24, L-6-24-24. Figure 2A,B.

*Cololejeunea gottschei (Stephani) Mizut.—**17**—354 m—Evergreen secondary tropical forest along sluggishly flowing stream in wide valley: partly shaded dry leaf of living shrub.—Associates: *Cololejeunea lanciloba*, *Drepanolejeunea* sp., *Leptolejeunea balansae*—L-6-9-24, L-6-40-24, L-6-46-24. Figure 1H.

*Cololejeunea lanciloba* Stephani—**9**, **17**—280, 354 m—Evergreen secondary tropical forests along a sluggishly flowing streams in wide valleys: partly shaded dry leaves of living shrubs—Associates: *Cololejeunea gottschei*, *Leptolejeunea balansae*—L-5-17-24, L-6-40-24.

*Cololejeunea raduliloba Stephani—**9**—280 m—Evergreen secondary tropical forest with tall bamboo understory along sluggishly flowing stream: partly shaded mesic boulder near the stream—L-5-19-24. Figure 2C.

*Cololejeunea spinosa (Horik.) Pandé et R.N. Misra—**9**—280 m—Evergreen secondary tropical forest with tall bamboo understory along a sluggishly flowing stream: partly shaded dry leaf of living shrub—L-5-18-24.

*Cololejeunea yakusimensis (S. Hatt.) Mizut.—**10**—308 m—Evergreen secondary tropical forest with tall bamboo understory along a sluggishly flowing stream: partly shaded dry bark of living standing tree trunk—Associates: *Lejeunea anisophylla*—Lao24070.

*Cyathodium cf. *cavernarum* Kunze—**20**, **22**—162, 170 m—Large karst limestone massif: mesic partly shaded fine soil covering limestone ledge in a very large cave with water-filled pool at the bottom—L-4-1-24, L-4-2-24, Lao24049—Comment: the plants in studied specimens are sterile and precise identification is impossible. Figure 2D. *Drepanolejeunea spicata* (Stephani) Grolle et R.L. Zhu—**18**—381 m—Evergreen secondary tropical forest along a sluggishly flowing stream in broad valley: an open and partly shaded dry leaf of living shrub.—Associates: *Leptolejeunea elliptica*—Lao24100.

*Dumortiera hirsuta (Sw.) Nees—**9**—280—Evergreen secondary tropical forest with tall bamboo understory along a sluggishly flowing stream: partly shaded moist clayish soil on steep slope to stream—Associates: *Bazzania* cf. *callida*, *Heteroscyphus coalitus*, *Pallavicinia levieri*—L-5-10-24, L-5-10a-24.

**Figure 3:**
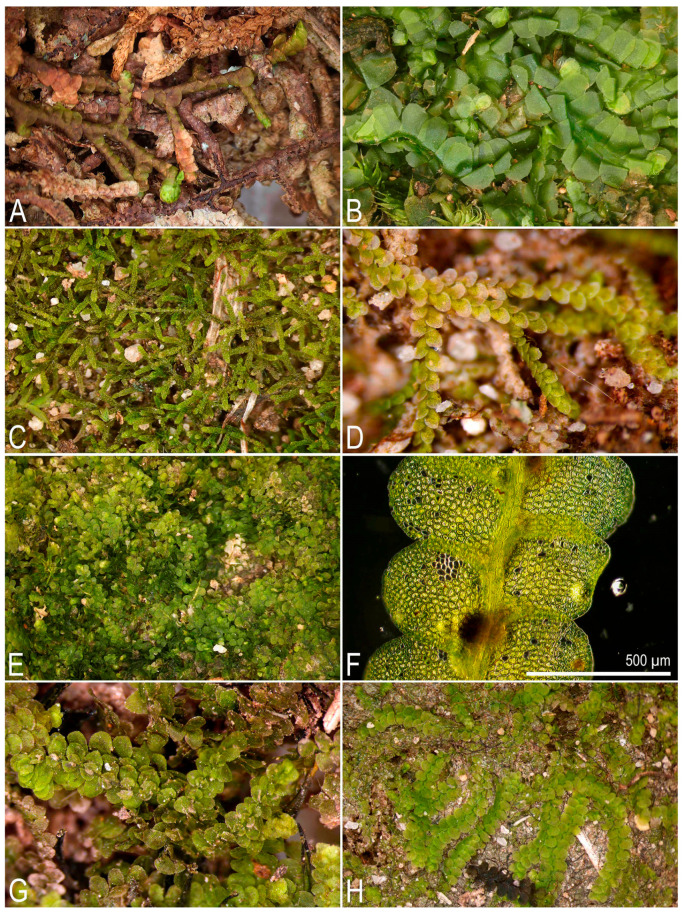
Some liverworts newly recorded in Laos (photos were made using upright compound and dissecting microscopes in the laboratory): (**A**) *Frullania brotheri* Steph.; (**B**) *Heteroscyphus zollingeri* (Gottsche) Schiffn.; (**C**) *Kurzia* cf. *gonyotricha* (Sande Lac.) Grolle; (**D**) *Lejeunea parva* (S. Hatt.) Mizut.; (**E**,**F**) *Lejeunea magohukui* Mizut.; (**G**) *Lejeunea obscura* Mitt.; (**H**) *Lejeunea pallidevirens* S. Hatt. (**A**) from L-6-18-24, (**B**) from L-6-3-24, (**C**) from L-6-14-24, (**D**) from L-6-25-24, (**E**,**F**) from L-6-13-24, (**G**) from L-5-1-24, (**H**) from L-1-10-24 (VBGI) (photos by V.A. Bakalin, 2024).

*Frullania brotheri Steph.—**17**—354 m—Evergreen secondary tropical forest along a sluggishly flowing stream in wide valley: partly shaded dry bark of a living tree branch—L-6-18-24. Figure 3A.

*Frullania gaudichaudii (Nees et Mont.) Nees et Mont.—**15**—317 m—Evergreen tropical forest in a broad stream valley: partly shaded mesic cliff near stream—L-1-2-24. Figure 2E,F.

*Frullania motoyana Steph.—**15**, **17**—317, 354—Evergreen tropical forest in a broad stream valley: open dry bark of living standing tree trunk. Evergreen secondary tropical forest along wide stream valley: open to partly shaded dry bark of living tree trunks and branches, partly shaded dry boulder near the stream—Associates: *Acrolejeunea recurvata*, *Schiffneriolejeunea polycarpa*—L-1-5-24, L-1-6-24, L-1-7-24, L-6-17-24, L-6-29-24, L-6-35-24, L-6-35a-24, L-6-36-24. Figure 2G,H.

*Frullania serrata Gottsche—**26**—973 m—No habitat data are provided in the 2023-11 label; based on satellite imagery, this is a rather dense evergreen forest along a wide stream valley—2023-11.

**Figure 4:**
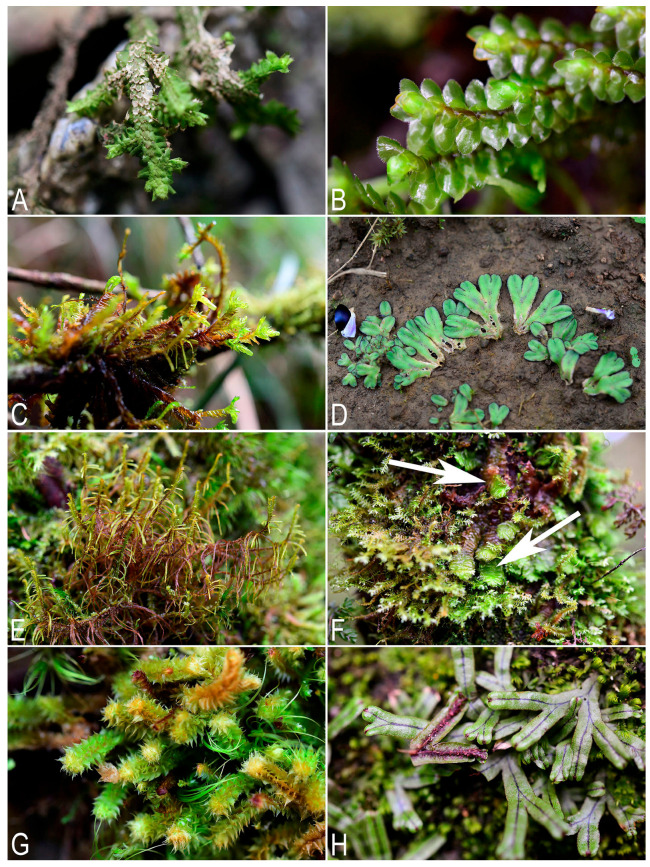
Some liverworts newly recorded in Laos (photos were made using a digital camera in natural habitats): (**A**) *Porella perrottetiana* (Mont.) Trevis.; (**B**) *Scapania ornithopoides* (With.) Waddell; (**C**) *Pleurozia subinflata* (Austin) Austin; (**D**) *Riccia* cf. *billardieri* Mont. et Nees ex Gottsche, Lindenb. et Nees; (**E**) *Herbertus armitanus* (Steph.) H.A. Mill.; (**F**) *Schistochila sciurea* (Nees) Schiffn. (larger shoots indicated by white arrows); (**G**) *Plicanthus hirtellus* (F. Weber) R.M. Schust.; (**H**) *Marchantia papillata* subsp. *grossibarba* (Steph.) Bischl. (photos by S.S. Choi, 2024–2025).

*Herbertus armitanus (Steph.) H.A. Mill.—**6**—2780 m—Evergreen montane rain forest on slope: open-to-partly shaded mesic rocks, branches and cliffs—Lao25129. Figure 4E.

*Heteroscyphus argutus* (Nees) Schiffn.—**8**, **9**—280, 333 m—Evergreen secondary tropical forest with tall bamboo understory along a sluggishly flowing stream: open mesic boulder near the stream, partly shaded mesic to moist clayish soil near streams and on steep slopes to them—Associates: *Pallavicinia levieri*, *Plectocolea horikawana*—L-5-13-24, L-5-20-24, L-5-7-24, Lao24053.

*Heteroscyphus coalitus* (Hook.) Schiffn.—**9**—280 m—Evergreen secondary tropical forest with tall bamboo understory along a sluggishly flowing stream: partly shaded moist clayish soil on steep slope to stream—Associates: *Asperifolia indosinica*, *Dumortiera hirsuta*, *Pallavicinia levieri*—L-5-10a-24.

*Heteroscyphus zollingeri (Gottsche) Schiffn.—**17**—354 m—Evergreen secondary tropical forest along sluggishly flowing stream in wide valley: partly shaded dry boulder and mesic clayish soil near the stream—L-6-3-24, L-6-4-24, L-6-8-24. Figure 3B.

*Kurzia cf. *gonyotricha* (Sande Lac.) Grolle—**17**—354 m—Evergreen secondary tropical forest along a sluggishly flowing stream in wide valley: partly shaded mesic clayish soil near the stream—L-6-14-24, L-6-7-24. Figure 3C.

*Lejeunea anisophylla Mont.—**10**, **16**—308, 379 m—Evergreen secondary tropical forests along a sluggishly flowing streams in wide valleys: partly shaded dry bark of living standing tree trunk; partly shaded dry fallen decaying tree trunk.—Associates: *Cololejeunea yakusimensis*, *Lopholejeunea* cf. *nigricans*—Lao24070, Lao24088.

*Lejeunea discreta Lindenb.—**23**, **26**—259, 973 m—No habitat data are provided in the label for 2023-39; based on satellite imagery, this is a rather dense evergreen forest along a broad stream valley. The L-3-2-24 was collected in a scattered evergreen forest covering limestone rocky outcrops, on partly shaded dry bark of living standing tree trunk—Associates: *Ceratolejeunea belangeriana*, *Spruceanthus semirepandus*—2023-39, L-3-2-24.

*Lejeunea magohukui Mizut.—**9**, **17**—280, 354 m—Evergreen secondary tropical forests along sluggishly flowing streams: partly shaded dry fallen decaying tree trunk and bark of living tree branch—L-5-15-24, L-6-13-24. Figure 3E,F.

*Lejeunea obscura Mitt.—**9**—280 m—Evergreen secondary tropical forest with tall bamboo understory along a sluggishly flowing stream: partly shaded dry bark of living standing tree trunk.—L-5-1-24. Figure 3G.

*Lejeunea pallidevirens S. Hatt.—**15**—317 m—Evergreen tropical forest in broad stream valley: partly shaded mesic root of the tree over cliff near the stream.—Associates: *Lopholejeunea* cf. *eulopha*—L-1-9-24, L-1-10-24. Figure 3H.

*Lejeunea parva (S. Hatt.) Mizut.—**17**, **24**—170, 354 m—Evergreen secondary tropical forest along a sluggishly flowing stream in wide valley: partly shaded dry bark of living tree trunk. Limestone rock outcrops covered with scattered evergreen forest: over the limestone outcrop—Associates: *Lopholejeunea* cf. *nigricans*—L-6-25-24, Lao24042. Figure 3D.

*Lejeunea cf. *sordida* (Nees) Nees—**10**—308 m—Evergreen secondary tropical forest with tall bamboo understory along a sluggishly flowing stream: partly shaded dry bark of living standing tree trunk—Lao24058.

*Lepidolejeunea bidentula (J.B. Jack et Stephani) R.M. Schust.—**16**—379 m—Evergreen secondary tropical forest along a sluggishly flowing stream in a wide valley: partly shaded dry bark of living standing tree trunk—Associates: *Cheilolejeunea trapezia*—Lao24085.

*Leptolejeunea balansae* Steph.—**11**, **16**, **17**—323–379 m—Evergreen tropical and evergreen secondary tropical forests in broad stream valleys: open and partly shaded dry leaves of living shrubs—Associates: *Cololejeunea gottschei*, *Cololejeunea lanciloba*—L-6-39-24, L-6-40-24, L-6-41-24, Lao24028, Lao24077, Lao24087, Lao24089.

*Leptolejeunea elliptica* (Lehm. et Lindenb.) Besch.—**15**, **17**, **18**—317–381m—Evergreen tropical and evergreen secondary tropical forests in broad stream valleys: partly shaded dry leaves of evergreen shrubs—Associates: *Drepanolejeunea spicata*—L-1-11-24, L-6-43-24, L-6-45-24, Lao24100.

*Lopholejeunea cf. *eulopha* (Taylor) Schiffn.—**15**, **17**—317, 354 m—Evergreen secondary tropical forest along a sluggishly flowing stream in a wide valley: partly shaded dry bark of living tree trunk. Evergreen tropical forest in a broad stream valley: mesic cliff near stream and mesic root of the tree over cliff near the stream—Associates: *Lejeunea pallidevirens*—L-1-1-24, L-1-9-24, L-6-27-24—Comment: the plants in all specimens are sterile and with not-so-large underleaves as it should be in the typical form of the species.

**Figure 5:**
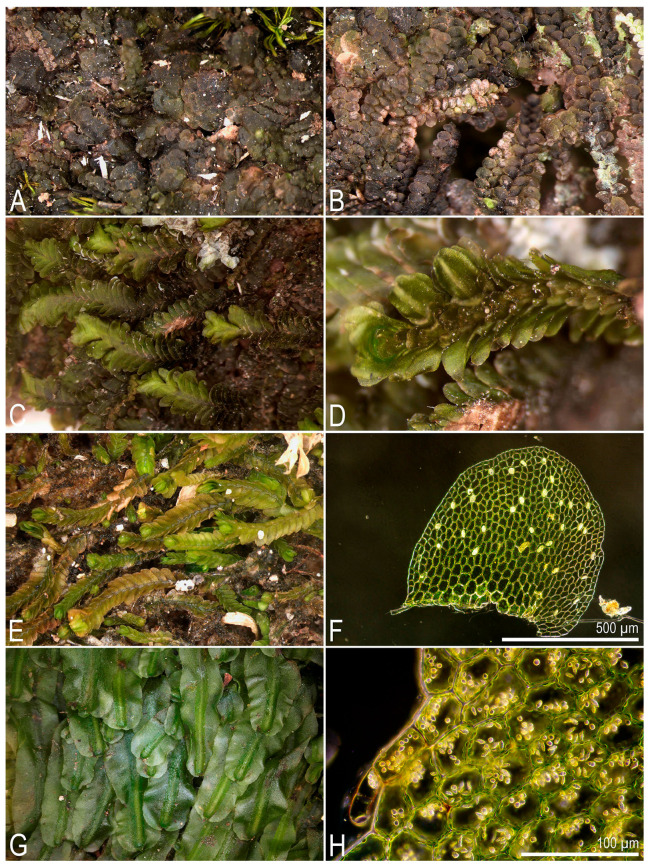
Some liverworts newly recorded in Laos (photos were made using upright compound and dissecting microscopes in the laboratory): (**A**) *Lopholejeunea nigricans* (Lindenb.) Steph. ex Schiffn.; (**B**) *Lopholejeunea subfusca* (Nees) Schiffn.; (**C**,**D**) *Mastigolejeunea indica* Stephani; (**E**) *Notoscyphus lutescens* (Lehm. et Lindenb.) Mitt.; (**F**) oil cells in the leaf of *Plectocolea tetragona* (Lindenb.) Amakawa; (**G**) *Pallavicinia levieri* Schiffn.; (**H**) oil bodies in marginal cells of *P. levieri* thallus. (**A**) from L-6-12-24, (**B**) from L-6-23-24, (**C**,**D**) from L-3-1-24, (**E**) from L-6-31-24, (**F**) from L-5-11-24, (**G**,**H**) from L-1-12-24 (VBGI) (photos by V.A. Bakalin, 2024).

*Lopholejeunea nigricans (Lindenb.) Steph. ex Schiffn.—**16**, **17**, **20**—170–379 m—Evergreen secondary tropical forest along a sluggishly flowing stream in a wide valley: partly shaded dry bark of living tree trunk. Large karst limestone massif covered with scattered evergreen forest: partly shaded dry bark of living tree branch—Associates: *Lejeunea anisophylla*, *Lejeunea parva*, *Mastigolejeunea indica*—L-4-6-24, L-6-12-24б L-6-25-24, Lao24088—Comment: the plants in most of the specimens are sterile and the identification of the majority of the specimens is questionable. Figure 5A.

*Lopholejeunea subfusca (Nees) Schiffn.—**16**, **17**—354, 379 m—Evergreen secondary tropical forest along a sluggishly flowing stream in a broad valley: partly shaded dry bark of living tree trunk.—Associates: *Lopholejeunea* sp.—L-6-23-24, Lao24081a. Figure 5B.

*Marchantia emarginata Reinw., Blume et Nees subsp. *tosana* (Steph.) Bischl.—**9**—280 m—Evergreen secondary tropical forest with tall bamboo understory along a sluggishly flowing stream: open moist sandy soil near the stream—L-5-16-24.

*Marchantia papillata subsp. *grossibarba* (Steph.) Bischl.—**2**—1123 m—No habitat data are provided in the labels; based on satellite imagery, this is a rather dense evergreen tropical forest along a stream valley—2023-1, 2023-3. Figure 4H.

*Mastigolejeunea indica Stephani—**20**, **21**, **23**—161–259 m—Large karst limestone massifs covered with scattered evergreen forest: partly shaded dry bark of living tree branch and tree trunks—Associates: *Lopholejeunea* cf. *nigricans*—L-3-1-24, L-4-5-24, L-4-6-24, Lao24045. Figure 5C,D.

*Mastigolejeunea repleta (Taylor) A. Evans—13, 25—301, 449 m—Limestone rock outcrops covered with scattered evergreen forest: over the limestone rocky outcrops. Evergreen tropical forest in wide stream valley: partly shaded dry bark of living standing tree trunk—Lao24036, Lao24043.

*Microlejeunea cf. *punctiformis* (Taylor) Steph.—**17**—354 m—Evergreen secondary tropical forest along a sluggishly flowing stream in a broad valley: partly shaded dry fallen tree trunk—L-6-16-24.

*Notoscyphus lutescens (Lehm. et Lindenb.) Mitt.—**17**—354 m—Evergreen secondary tropical forest along a sluggishly flowing stream in a broad valley: partly shaded dry boulder near the stream—L-6-31-24. Figure 5E.

*Pallavicinia levieri Schiffn.—**9**, **15**, **17**, **18**—280–381 m—Evergreen tropical and secondary tropical forests along broad stream valleys: partly shaded dry boulder near the stream, open and partly shaded moist cliffs near the stream, partly shaded moist clayish soil on steep slope to stream—Associates: *Bazzania* cf. *callida*, *Dumortiera hirsuta*, *Heteroscyphus argutus*, *Heteroscyphus coalitus*, *Riccardia* cf. *graeffei*, *Riccardia graeffei*—L-1-12-24, L-1-13-24, L-5-10-24, L-5-10a-24, L-5-3-24, L-5-7-24, L-6-5-24, L-6-6-24, Lao24101a. Figure 5G,H.

*Plagiochila beddomei Steph.—**4**—1805 m—No habitat data are provided in the label for 2023-5; based on satellite imagery, this is a very scattered secondary forest on a gentle slope—Associates: *Bazzania siamensis*—2023-5, 2023-24—Comment: the specimen 2023-05 has fragmenting leaves in some plants and the identification is questionable for it.

*Plagiochila cf. *fordiana* Stephani—**26**—973 m—No habitat data are provided in the label for 2023-33; based on satellite imagery, this is a rather dense evergreen forest along a wide stream valley—2023-33.

*Plagiochila junghuhniana Sande Lac.—**26**—973 m—No habitat data are provided in the labels for the specimens; based on satellite imagery, this is a a rather dense evergreen forest along a wide stream valley—2023-10, 2023-9.

*Plagiochila peculiaris Schiffn.—**26**—973 m—No habitat data are provided in the label for 2023-28; based on satellite imagery, this is a rather dense evergreen forest along a wide stream valley—2023-28.

*Plagiochila vexans Schiffn. ex Steph.—**4**—1805 m—No habitat data are provided in the label for 2023-22; based on satellite imagery, this is a very scattered secondary forest on a gentle slope.—2023-22.

**Figure 6:**
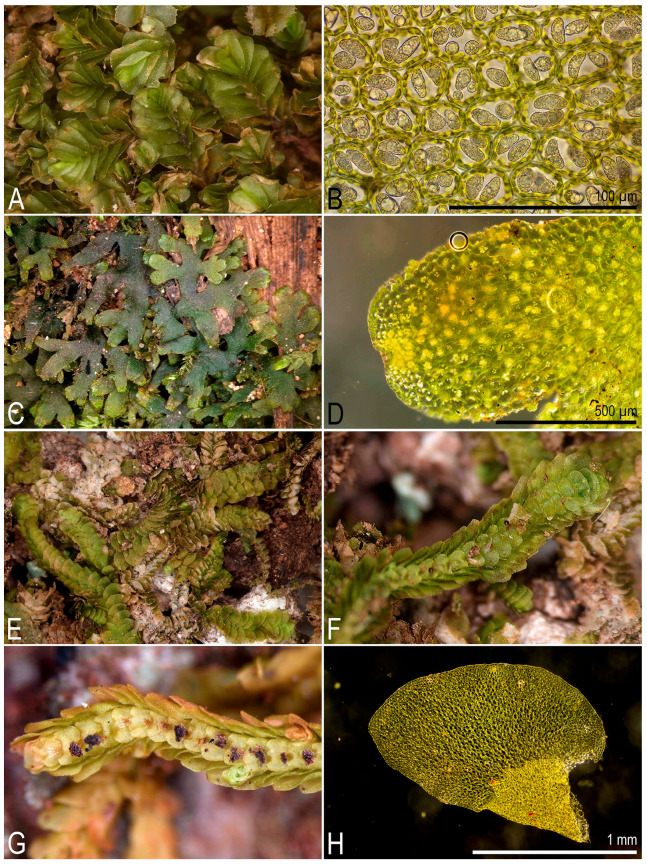
Some liverworts newly recorded in Laos (photos were made using upright compound and dissecting microscopes in the laboratory): (**A**) *Plectocolea horikawana* Amakawa; (**B**) oil bodies in midleaf cells of *P. horikawana*; (**C**) *Riccardia graeffei* (Stephani) Hewson; (**D**) oil bodies in cells of the shoot apex of *R. graeffei*; (**E**,**F**) *Schiffneriolejeunea polycarpa* (Nees) Gradst.; (**G**) *Schiffneriolejeunea pulopenangensis* (Gottsche) Gradst.; (**H**) leaf of *S. pulopenangensis*, lobule has three teeth. (**A**,**B**) from L-5-13-24, (**C**,**D**) from L-5-9-24, (**E**,**F**) from L-1-4-24, (**G**,**H**) from L-6-28-24 (VBGI) (photos by V.A. Bakalin, 2024).

*Plectocolea horikawana Amakawa—**9**—280 m—Evergreen secondary tropical forest with tall bamboo understory along a sluggishly flowing stream: open mesic boulder near the stream—Associates: *Heteroscyphus argutus*—L-5-13-24. Figure 6A,B.

*Plectocolea tetragona (Lindenb.) Amakawa—**9**, **17**—280, 354 m—Evergreen secondary tropical forests along sluggishly flowing streams in broad valleys: partly shaded mesic clayish soil near the stream, partly shaded moist clayish soil on steep slope to stream—Associates: *Plectocolea truncata*—L-5-11-24, L-6-10-24. Figure 5F.

*Plectocolea truncata* (Nees) Bakalin—**1**, **9**, **10**, **17**—280–354, 1384 m—Evergreen secondary tropical forests along broad stream valleys and evergreen forest on the ridgeline. Partly shaded moist clayish soil on steep slope to stream, open moist cliff near the stream. The ecology for mountain ridge is not indicated—Associates: *Pallavicinia levieri*, *Plectocolea tetragona*, *Riccardia graeffei*—2023-13, 2023-14, 2023-15, 2023-16, L-5-3-24, L-5-11-24, L-5-8-24, L-6-10-24, Lao24069.

*Pleurozia subinflata (Austin) Austin—**5**, **6**—2690, 2780 m—Evergreen montane rain forest on slope: open to partly shaded mesic tree trunks, branches and cliffs—Lao25141, Lao25143, Lao25229, La25258. Figure 4C.

*Plicanthus hirtellus (F. Weber) R.M. Schust.—**5**, **6**—2690, 2780 m—Evergreen montane rain forest on slope: open to partly shaded mesic rocks, branches and cliffs—Lao25103, Lao25124, Lao25184. Figure 4G.

*Porella acutifolia* (Lehm. et Lindenb.) Trevis.—**7**—1270 m—Evergreen tropical forest on mountain slope: open to partly shaded mesic, rarely moist, limestone cliffs and tree trunks—Lao25015, Lao25031.

*Porella perrottetiana* (Mont.) Trevis.—**7**—1270 m—Evergreen tropical forest on mountain slope: open to partly shaded mesic, rarely moist, limestone cliffs and tree trunks—Lao25017. Figure 4A.

*Ptychanthus striatus* (Lehm. et Lindenb.) Nees—**3**, **26**—948, 973 m—No habitat data are provided in the label for the specimens; based on satellite imagery, this is a rather dense evergreen forest along wide and narrow stream valleys—2023-12, 2023-18, 2023-19, 2023-21, 2023-29, 2023-35.

*Riccardia graeffei (Stephani) Hewson—**9**, **17**—280, 354 m—Evergreen secondary tropical forests along sluggishly flowing streams in broad valleys: open moist cliff near the stream, partly shaded moist clayish soil on steep slope to stream, partly shaded moist humus near the stream, partly shaded dry boulder near the stream—Associates: *Pallavicinia levieri*, *Plectocolea* cf. *truncata*—L-5-3-24, L-5-5-24, L-5-9-24, L-6-5-24. Figure 6C,D.

*Riccia cf. *billardieri* Mont. et Nees ex Gottsche, Lindenb. et Nees—**20**—170 m—Agricultural lands surrounding large karst limestone massif: moist clayish soil in agricultural land—L-4-3-24, L-4-4-24—Comment: the studied specimens are sterile and their identity is questionable. Figure 4D.

*Scapania ligulata Steph.—**5**—2690 m—Evergreen montane rain forest on slope: open to partly shaded moist to wet cliffs, including those near streams and in the streambeds—Lao25186.

*Scapania ornithopoides (With.) Waddell—**5**, **6**—2690, 2780 m—Evergreen montane rain forest slope of mountain: open, rarely partly shaded, moist to wet cliffs, including those near streams—Lao25066, Lao25082, Lao25090, Lao25099, Lao25102, Lao25109, Lao25116, Lao25138, Lao25191, Lao25249. Figure 4B.

*Schiffneriolejeunea polycarpa (Nees) Gradst.—**12**, **15**, **17**—317–470 m—Evergreen secondary tropical forest along wide stream valleys: partly shaded dry boulder near the stream and dry to mesic bark of living standing tree trunks—Associates: *Cheilolejeunea trifaria*, *Frullania motoyana*—L-1-3-24, L-1-4-24, L-1-4a-24, L-2-1-24, L-2-2-24, L-6-35-24, L-6-35a-24, L-6-36-24, L-6-37-24. Figure 6E,F.

*Schiffneriolejeunea pulopenangensis (Gottsche) Gradst.—**17**—354 m—Evergreen secondary tropical forest along a sluggishly flowing stream in wide valley: partly shaded dry bark of living tree trunk, dry boulder near the stream—L-6-28-24, L-6-36-24. Figure 6G,H.

*Schistochila aligera (Nees et Blume) J.B. Jack et Stephani—**26**—973 m—No habitat data are provided in the label for 2023-39; based on satellite imagery, this is a rather dense evergreen forest along a wide stream valley—2023-7.

*Schistochila sciurea (Nees) Schiffn—**6**—2780 m—Evergreen montane rain forest on slope: partly shaded mesic tree—Lao25169. Figure 4F.

*Spruceanthus planiusculus (Mitt.) X.Q. Shi, R.L. Zhu et Gradst.—**12**, **13**, **19**—184–470 m—Evergreen tropical forest in wide stream valley: partly shaded dry bark of living standing tree trunks—L-2-3-24, Lao24029, Lao24039.

*Spruceanthus semirepandus* (Nees) Verd.—**4**, **26**—973, 1805 m—No habitat data are provided in the label for 2023-39; based on satellite imagery, this is a rather dense evergreen forest along a wide stream valley and very scattered secondary forest on a gentle slope—Associates: *Ceratolejeunea belangeriana*—2023-26, 2023-27, 2023-39, 2023-6.

*Thysananthus aculeatus Herzog—**14**—313 m—Evergreen tropical forest in wide stream valley: partly shaded dry bark of living standing tree trunk—Lao24015.

*Thysananthus spathulistipus* (Reinw.) Lindenb.—**15**—317 m—Evergreen tropical forest in wide stream valley: partly shaded moist bark of living tree branch—L-1-8-24.

## 3. Discussion

The Section 2 lists 22 genera new to the liverwort flora of Laos. Without exception, these genera should have been found in the country, if its geographic location and landscape diversity had been taken into account. The list of these newly recorded genera once again demonstrates how little we know about the liverwort flora of Laos. Moreover, while some newly recorded genera (such as *Schistochila* and *Pleurozia*) require merely humid habitats, for others, such as *Lopholejeunea*, the only explanation for the fact that they were not known before is a critical lack of research.

The same applies to the number of newly recorded taxa for the liverwort flora of Laos: the 62 species of the total 76 observed are newly recorded for the country. Since approximately 80% of the total collected species are newly recorded to the country, we cannot expect to reach a plateau in the “knowledge curve” [26,51] and our collection is merely a random sample of the flora. The study areas were determined based on the feasibility of conducting pteridological studies in the country, and hepaticologists worked mostly at lower elevations. This affected the representation of the number of known species by elevation. In this regard, the distribution is as follows: for the 0–500 m a.s.l. range, we identified 57 species, for the 500–1000 m a.s.l. range—9 species, for 1000–1500 m a.s.l. range—4 species, for 1500–2000 m a.s.l. range—4 species and 6 species above 2500 m a.s.l.

We reiterate that the research conducted by hepaticologists was conducted at a relatively taxonomically unpromising elevation. The studied low-elevation belt is characterized by drier climate than areas at higher elevations, and its communities have been heavily altered by anthropogenic impacts. All the forests we visited at low elevations are clearly secondary in origin. In this regard, it is meaningful to examine the list of 57 species we provide for elevations below 500 m a.s.l., in order to understand which taxa can survive in such clearly unfavorable conditions. In this regard, a comparison with the liverwort flora of Vietnam is appropriate. Taking into account the most recently published checklists [21,22] and subsequent additions [23,24,25,26,27,28,29,30,31,32,33,34,35,36,37,38], approximately 600 species are known in the flora of Vietnam (the exact number is difficult to determine, as the status of some taxa is unclear, and some taxa are likely based on erroneous identifications). In Cambodia, 211 species are currently known [28,46,47], which, as we noted before [47], is still significantly lower than our expectations. However, this number is higher than the 128 species known in Laos. Therefore, if a species is listed as unknown in Vietnam, we indicate whether it is known in Cambodia.

Only seven species were found to be unknown in Vietnam. Four of them, *Bazzania callida*, *Ceratolejeunea belangeriana*, *Frullania gaudichaudii* and *Lejeunea* cf. *sordida*, are known in Cambodia. It is likely that all of them will be found in Vietnam in the future, once the taxonomically poor floras of the lower elevations have been more thoroughly studied there. The fifth species, *Plectocolea horikawana*, is unknown in both Vietnam and Cambodia. However, in this case, there is no certainty about the identification; it is possible that the species belongs to an as-yet-undescribed taxon, although we did not see any clear morphological differences from the typical *P. horikawana* from Japan. The absence of two more species from the genus *Schiffneriolejeunea* in Vietnam and Cambodia is a puzzle. *Schiffneriolejeunea polycarpa* is known in adjacent areas from China and Thailand, and *S. pulopenangensis* is widely distributed in SE Asia and also northward in China [52]. We hypothesize that both species may be found both in Vietnam and Cambodia.

According to the maps in the previously published Laos liverwort checklist [48] (Figure 1 and Figure 2 in the cited paper), in the most promising (by analogy with Vietnam and Thailand) Northern Indochina Subtropical Forests ecoregion, data are available only for Houaphanh Province, where five species are known. The Luang Prabang Montane Rain Forests ecoregion has data only for Xiangkhouang Province, where 26 species are known. Moreover, this ecoregion covers both the highest and the second-highest points of the Annamite Range, and 26 known species are certainly very few. The Central Indochina Dry Forests ecoregion, interspersed with the Southeastern Indochina Dry Evergreen Forests ecoregion, has data for Salavan (a single species) and Champasak (40 species) Provinces. It should be noted that the Northern Annamites Rain Forests ecoregion, potentially promising for liverwort diversity, has no data on its liverwort inhabitants.

The breakdown of species by province in our materials is provided in the Results section. Thus, completely new information was collected for five provinces: Phongsaly, Oudomxay, Vientiane, Bolikhamsai, and Khammouane. In total, at least some data is currently available for nine provinces. Considering that there are currently 17 provinces in Laos (https://en.wikipedia.org/wiki/Provinces_of_Laos, accessed on 10 December 2025), this number is certainly not sufficient to provide a clear understanding of the intra-country liverwort distribution patterns as well.

Regarding future research, it can be assumed that, similar to Vietnam [26], the greatest number of species can be collected in the upper mountain belts. The northern part of the country is contiguous with the northern spurs of the Hengduan Range, and a number of Sino-Himalayan species may be found there. Exploration of the Annamite Range, which separates Vietnam and Laos, may be equally, if not more, promising. The range’s highest point, Phou Bia Mt. (2820 m a.s.l.), is located within Laos. Due to restrictions imposed by the large number of unexploded ordnance, this mountain has been closed to research visits for over 40 years, and non-Lao citizens have not been permitted to visit it. However, a program to establish a tourist route there has recently been developed and initiated, which should noticeably improve accessibility to the communities (https://en.wikipedia.org/wiki/Phou_Bia, accessed on 10 December 2025). In 2025, S.S.C. and I.C.H. visited the mountain, but they could collect only along the trail and only a few collections were made. The second-highest peak in the Annamite Range, Mount Phu Xai Lai Leng, is located on the border of Laos and Vietnam and reaches 2720 m above sea level. We visited this mountain from the Vietnamese side in 2023 (the survey results have not been published, except for a description of the discovery of a liverwort genus, *Gottschelia*, new to Indochina [29]). Since our research extended as far as the Lao–Vietnamese border, we can confirm that equally rich liverwort areas are also found in Laos, which borders Vietnam. Such locations along the Vietnam–Laos border deserve particular attention in the future.

## 4. Materials and Methods

Laos is a landlocked country located in the central part of the Indochina Peninsula. It lies entirely south of the Tropic of Cancer and extends from approximately 22°30′ N to 15°50′ N, covering 236,800 square kilometers (being somewhere between Romania and Guyana in size). A brief, yet adequate for our purposes, description of the country’s climate and biomes was provided by Söderström et al. [48]. Therefore, we present here only the data pertaining to the provinces we visited. In 2023, 2024, and 2025, four expeditions were conducted under the auspices of the Korean National Institute of Biological Resources, primarily to study pteridophytes. During the first two, several specimens were collected by pteridologist I.C.H. in the north of the country and in Champasak Province in the south, while in the third (May 2024), the collections were assembled by hepaticologists V.A.B. and S.S.C. in the central part of the country, at lower elevations, in relatively dry secondary forests. The fourth expedition was carried out by I.C.H. and S.S.C. in 2025 and took place along a newly opened trail to the highest point of the Annamite Range (Phou Bia Mt.), but very few specimens were collected there due to administrative restrictions.

**Figure 7:**
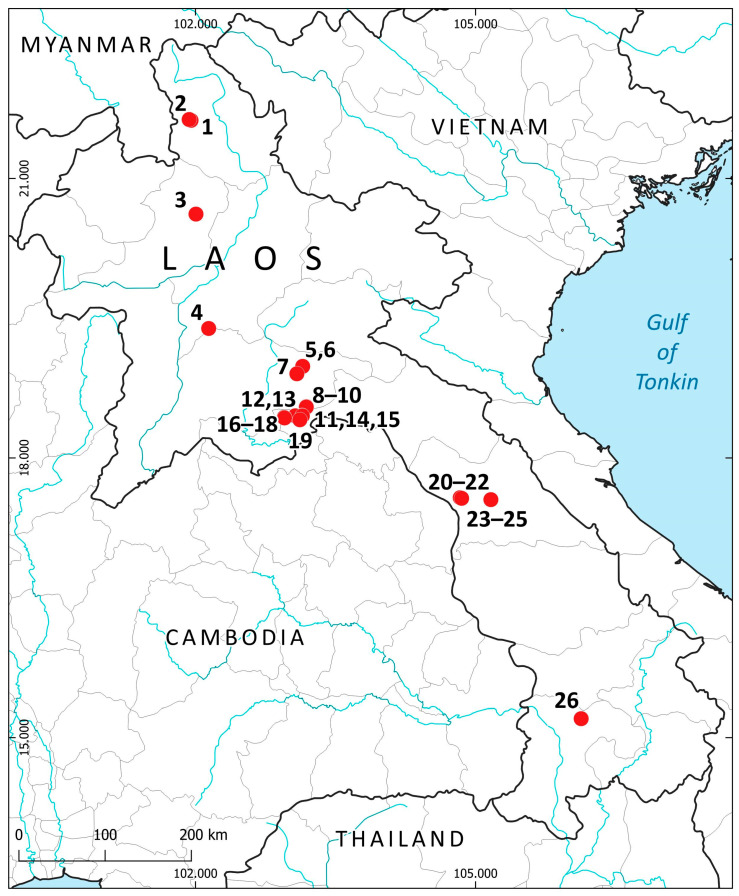
Collecting localities in Laos (in accordance with Table 1 and the list of species).

**Figure 8:**
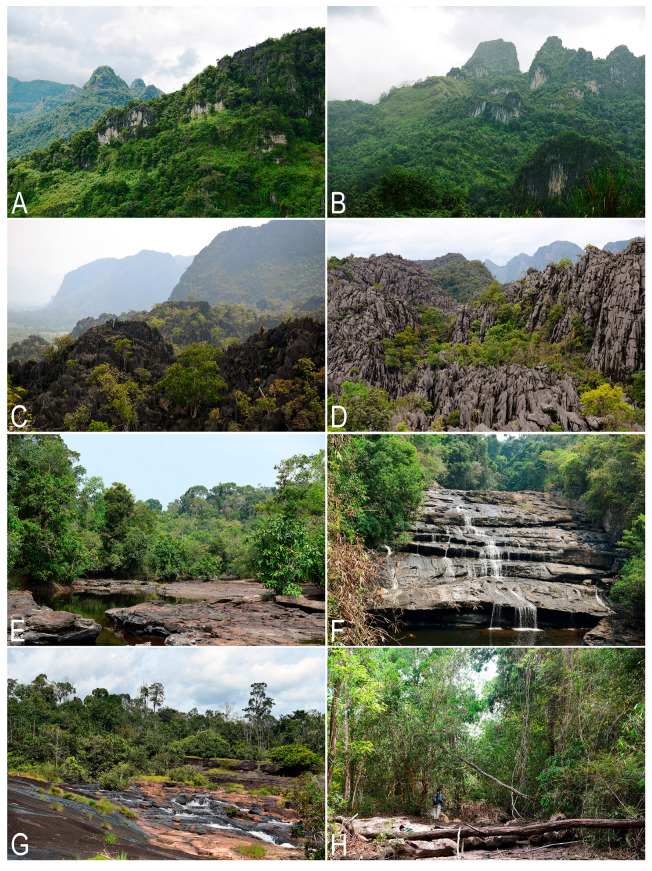
Landscapes of field trips in Laos: (**A**,**B**) Phou Bia Mt. in Xaisomboun Province; (**C**,**D**) limestone rock outcrops with scattered evergreen forest in Khammouane Province; (**E**) an evergreen tropical forest in a wide stream valley in Bolikhamsai Province; (**F**) Tadxai waterfall in Bolikhamsai Province; (**G**) Tad Phase waterfall in Bolikhamsai Province; (**H**) an evergreen secondary tropical forest along a sluggishly flowing stream in a wide valley in Bolikhamsai Province (photos by S.S. Choi, 2024–2025).

**Figure 9:**
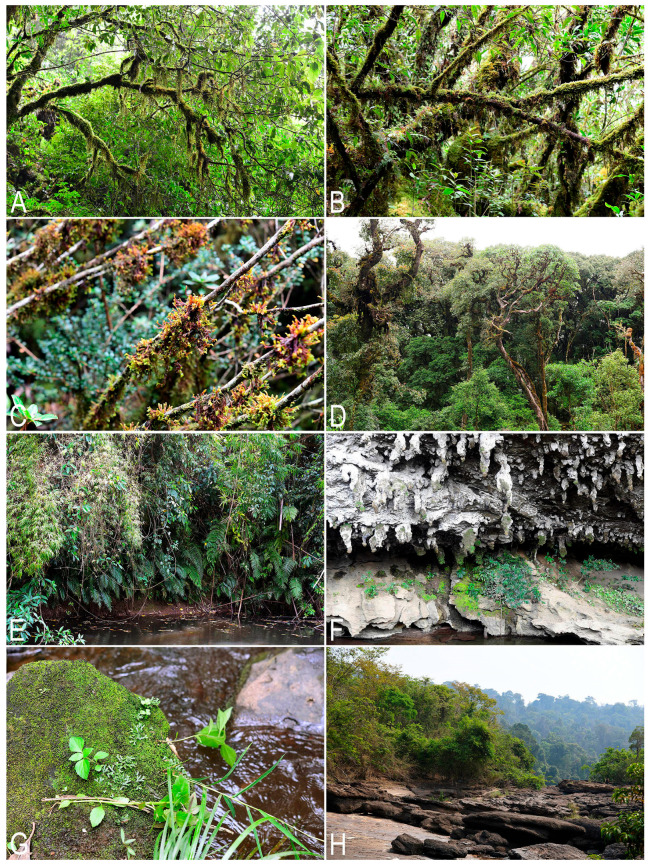
Some habitats of liverworts in Laos: (**A**,**B**) tree branches with hanging mixed mosses and liverworts in an evergreen montane rainforest at Mt. Phou Bia in Xaisomboun Province; (**C**) tree branches with *Pleurozia subinflata* (Austin) Austin on Mt. Phou Bia in Xaisomboun Province; (**D**) tree bark on Mt. Phou Bia in Xaisomboun Province; (**E**) shaded wet soil in an evergreen secondary tropical forest along a sluggishly flowing stream in Xaisomboun Province; (**F**) large karst limestone massif covered with a scattered evergreen forest with caves in Khammouane Province; (**G**) wet rocks along a sluggishly flowing stream in Xaisomboun Province; (**H**) rocks in the valley of Phou Khao Khouay National Park in Xaisomboun Province (photos by S.S. Choi, 2024–2025).

The vegetation, landscape and geographical position are summarized in Table 1. Figure 7 shows the liverwort-collecting localities in accordance with Table 1. Figure 8 shows some landscapes where liverworts were collected; Figure 9—some liverwort habitats.

As can be seen from Table 1 (the territorial distribution of collection sites), most of the areas above 900 m above sea level were visited by a pteridologist (I.C.H.), who collected liverworts only occasionally and irregularly. Another expedition to the higher elevations was conducted in 2025 by I.C.H. and S.S.C. in Phou Bia Mt. (the highest mountain in Laos). The area was previously strictly off-limits for foreigners due to the large number of unexploded bombs in the area. However, this year (2025), a new trail was established, although with strict limitations on access to areas beyond the trail. Only a few specimens were collected and processed from there, but this information provides valuable data on the highest elevations of the Annamite Range. Therefore, there are few specimens from higher elevations, but most of the species collected there were not found at lower elevations.

Therefore, hepaticologists in our team mostly visited areas below 500 m above sea level, i.e., in dry and strongly anthropogenically modified areas. All forest communities visited there are highly modified secondary evergreen forests, typically with a dense canopy of tall bamboo in the understory. The advantage of collecting at lower elevations is that hepaticologists typically do not work in such apparently unpromising areas, and the data obtained indicate which species can survive in such unfavorable conditions.

During collection, the geographic coordinates and altitude above sea level (according to GPS receiver data) were recorded for all gathered specimens. For the collections by S.S.C. and V.A.B., the substrate, community type, degree of moisture and shading (based on visual assessment, without the use of special equipment) were also recorded. All specimens collected by V.A.B. were delivered to the laboratory alive, which also made it possible to document the oil bodies in the cells of the collected plants. Other specimens were dried soon after collection. The specimens collected by V.A.B. were studied in the Laboratory of Cryptogamic Biota of the Botanical Garden-Institute of the Russian Academy of Sciences, using an Olympus CX43 (Olympus Corporation, Tokyo, Japan) (upright compound microscope) and an Olympus SZX16 (Olympus Corporation, Tokyo, Japan) (dissecting microscope). The specimens collected by S.S.C. and I.C.H. were studied at the facilities of the Korean National Institute of Ecology (Seocheon), using an upright compound microscope LEICA DM 2500 (Leica Microsystems, Wetzlar, Germany) and a dissecting microscope LEICA M205C (Leica Microsystems, Wetzlar, Germany). Accordingly, the specimens collected by V.A.B. are preserved in the VBGI herbarium, while those of S.S.C. and I.C.H. are housed in the JNU herbarium (although duplicates of many specimens were donated to VBGI). If necessary, the collected specimens can be requested from these herbaria.

The provided checklist consists of the names arranged alphabetically with nomenclature according to the World Liverwort Checklist [50]: the genera first and then species within the genus. The exception is made for the genus *Plectocolea* (Mitt.) Mitt., which is treated here as a genus separate from *Solenostoma* (the same as with the list of Cambodian liverworts [47]). Each species is annotated according to the following scheme: (1) accepted species name, (2) collection locality numbers according to Table 1 and Figure 7, provided in bold font, (3) altitudinal distribution of the taxon (if only two elevations are known, they both are provided, if more, they are united as range using a dash, but if the difference in elevation between adjacent localities is more than 300 m, the altitudinal measurements are listed in full), (4) ecological conditions of occurrence (where available), (5) associated species (where available), (6) list of field collection numbers, and (7) comments in some cases. Subdivisions of the annotation are separated by a long dash. Specimen codes are given in accordance with the collection authors’ preferences and begin with ‘L-’ for V.A.B., with ‘Lao’ for S.S.C., and with ‘2023-’ for I.C.H. The newly recorded taxa for the country are marked with an asterisk.

## 5. Conclusions

The liverwort flora of Laos remains vastly understudied. The currently known number of species (128) falls short of what would be expected for a country with such diverse landscapes. However, one should not expect the same high diversity as in neighboring Vietnam or Thailand, as Laos is a much drier country and also has a more limited latitudinal range. Most of the collected species are newly recorded to Laos, and 22 genera of liverworts are also newly recorded to the country. Since the study by hepaticologists was conducted mostly at lower elevations, the number of species collected is relatively small. However, an interesting result is that liverworts are nevertheless present (57 species were recorded), and often in considerable numbers, in completely modified communities. Interestingly, even under these conditions, seven of the reported species collected at lower elevations are unknown in Vietnam. Although four of them are known in Cambodia and two more in Thailand, these findings were largely expected. Further research should likely be focused on the upper elevations in the north and east of the country, where there is a high probability of discovering a large number of liverwort species new to Laos.

## Figures and Tables

**Table 1:** Description of collecting localities treated in the present paper.

No.	Coordinates	Altitude, m a.s.l.	Province	Geographic Description	Collector and Collection Date	Biomes and Ecoregions, Where Collections Were Made
1	21.62397° N 101.95253° E	1384	Phongsaly	Boun Neua District, Ban Hoaylek. Mountain ridge	I.C. Hwang 6 October 2023	North Indochina Subtropical Forests
2	21.63322° N 101.93225° E	1123	Phongsaly	Boun Neua District, Ban Hoaylek	I.C. Hwang 6 October 2023	
3	20.61761° N 102.00540° E	948	Oudomxay	Houaylin. Muang Tai waterfall	I.C. Hwang 3 October 2023	
4	19.38966° N 102.14151° E	1805	Vientiane	Ban Pha Lam Phen, near Kasi Viewpoint	I.C. Hwang 2 October 2023	Luang Prabang Montane Rain Forest
5	18.98339° N 103.14758° E	2690	Xaisomboun	Anouvong, Ban Mouang Cha, Mt. Phou Bia. Roadside	S.S. Choi 23 August 2025	
6	18.982806° N 103.14872° E	2780	Xaisomboun	Anouvong, Ban Mouang Cha, Mt. Phou Bia. Mountain slope	S.S. Choi 22 August 2025	
7	18.90328° N 103.08647° E	1270	Xaisomboun	Anouvong, Neng YeLor viewpoint. Limestone rock outcrops with evergreen forest	S.S. Choi 21 August 2025	
8	18.55108° N 103.18822°E	333	Xaisomboun	Lavec Commune. Evergreen secondary tropical forest with tall bamboo understory along sluggishly flowing stream	S.S. Choi 7 May 2024	
9	18.54568° N 103.18860° E	280	Xaisomboun	Lavec Commune. Evergreen secondary tropical forest with tall bamboo understory along sluggishly flowing stream	V.A. Bakalin 7 May 2024	
10	18.54547° N 103.18733° E	308	Xaisomboun	Lavec Commune. Evergreen secondary tropical forest with tall bamboo understory along sluggishly flowing stream	S.S. Choi 7 May 2024	
11	18.45447° N 103.14211° E	323	Bolikhamsai	Hat Khay Commune. Evergreen tropical forest in wide stream valley	S.S. Choi 4 May 2024	
12	18.45229° N 103.06925° E	470	Bolikhamsai	Hat Khay Commune. Evergreen tropical forest in wide stream valley	V.A. Bakalin 4 May 2024	
13	18.45217° N 103.06939° E	449	Bolikhamsai	Hat Khay Commune. Evergreen tropical forest in wide stream valley	S.S. Choi 4 May 2024	
14	18.45200° N 103.14472° E	313	Bolikhamsai	Hat Khay Commune. Evergreen tropical forest in wide stream valley	S.S. Choi 4 May 2024	
15	18.45196° N 103.14475° E	317	Bolikhamsai	Hat Khay Commune. Evergreen tropical forest in wide stream valley	V.A. Bakalin 4 May 2024	
16	18.43653° N 102.95147° E	379	Bolikhamsai	Thaphabath Commune. Evergreen secondary tropical forest along sluggishly flowing stream in wide valley	S.S. Choi 8 May 2024	
17	18.43588° N 102.95078° E	354	Bolikhamsai	Thaphabath Commune. Evergreen secondary tropical forest along sluggishly flowing stream in wide valley	V.A. Bakalin 8 May 2024	
18	18.42906° N 102.95406° E	381	Bolikhamsai	Thaphabath Commune. Evergreen secondary tropical forest along sluggishly flowing stream in wide valley.	S.S. Choi 8 May 2024	
19	18.40944° N 103.11842° E	184	Bolikhamsai	Hat Khay Commune. Evergreen tropical forest in wide stream valley	S.S. Choi 4 May 2024	
20	17.57441° N 104.84003° E	170	Khammouane	Ban Nahouangoua Commune. Large karst limestone massif covered with scattered evergreen forest with caves, surrounded by agricultural lands	V.A. Bakalin 6 May 2024	Northern Kharat Plateau Moist Deciduous Forests
21	17.57328° N 104.83611° E	161	Khammouane	Ban Nahouangoua Commune. Large karst limestone massif covered with scattered evergreen forest with caves, surrounded by agricultural lands	S.S. Choi 6 May 2024	
22	17.56617° N 104.85153° E	162	Khammouane	Ban Nahouangoua Commune. Large karst limestone massif covered with scattered evergreen forest with caves, surrounded by agricultural lands	S.S. Choi 6 May 2024	
23	17.55285° N 105.16443° E	259	Khammouane	Phu Kheng Commune. Limestone rock outcrops with scattered evergreen forest	V.A. Bakalin 5 May 2024	
24	17.55272° N 105.16417° E	170	Khammouane	Phu Kheng Commune. Limestone rock outcrops with scattered evergreen forest	S.S. Choi 5 May 2024	
25	17.55186° N 105.16497° E	301	Khammouane	Phu Kheng Commune. Limestone rock outcrops with scattered evergreen forest	S.S. Choi 5 May 2024	
26	15.20289° N 106.13217° E	973	Champasak	Paksong, Vang Ngao River, Tad Champee Waterfall	I.C. Hwang 31 July 2023	Central Indochina Dry Forests

## Data Availability

The original contributions presented in this study are included in the article. Further inquiries can be directed to the corresponding authors.

## Abbreviations

The following abbreviations are used in this manuscript:

FEB RAS	Far Eastern Branch of the Russian Academy of Sciences
VBGI	Herbarium of the Botanical Garden-Institute FEB RAS, Vladivostok
JNU	Herbarium of the Chonbuk National University, Chonju
